# A Rare Case of Vasculitis Patched Necrosis of Cecum due to Behçet's Disease

**DOI:** 10.1155/2017/1693737

**Published:** 2017-05-18

**Authors:** Ehsan Shahverdi, Mehdi Morshedi, Faraneh Oraei-Abbasian, Maryam Allahverdi Khani, Roya Khodayarnejad

**Affiliations:** ^1^Blood Transfusion Research Center, High Institute for Research Center and Education in Transfusion Medicine, Tehran, Iran; ^2^Blood and Cancer Research Center, MAHAK Pediatric Cancer Treatment and Research Center, Tehran, Iran; ^3^Department of Surgery, Baqiyatallah University of Medical Sciences, Tehran, Iran; ^4^Mashhad University of Medical Sciences, Mashhad, Iran; ^5^Department of Medicine, Najafabad Branch, Islamic Azad University, Najafabad, Iran; ^6^Tehran Medical Sciences Branch, Islamic Azad University, Tehran, Iran

## Abstract

Isolated cecal necrosis is a rare form of acute ischemic colitis and a rare cause of surgical abdomen. Behçet's disease is a multisystemic autoimmune condition which can induce vasculitis. This can result in cecal necrosis while disease involves colon vessels. We describe a patient with complicated Behçet's disease and ischemic colitis admitted to our hospital. Patient was a 62-year-old female with more accompanying diseases. Histopathologic findings confirmed the diagnosis of ischemic colitis and regarding patient's vision problem and skin lesions, rheumatologic tests were performed which were positive for HLAB5 and HLAB51 suggestive of Behçet's disease; the patient was improved with surgery. Abdominal pain can indicate a disease with vascular involvement like Behçet's disease, especially in the presence of other clinical findings suggestive of the disease including blurred vision and skin lesions. An abdominal computerized tomography (CT) scan is very diagnostic in the same patients.

## 1. Introduction

Ischemic colitis is a vascular disorder of the colon which causes rectal bleeding and abdominal pain in elderly patients [1]. It mostly develops in relation to atherosclerosis and low blood flow of colon arteries [2]. Isolated cecal necrosis is a rare form of acute colonic ischemia and a rare cause of surgical abdomen [3]. It generally presents with right lower quadrant pain and is clinically shown similar to that of acute appendicitis. These patients might have one or more accompanying diseases [3–5]. Among these diseases, heart failure and chronic renal failure are at the top of the list. In this case report, we describe a patient with partial cecal necrosis due to ischemic colitis with vascular involvement in the background of Behçet's disease. Behçet's disease is a systemic inflammatory disorder with unknown etiology. It commonly occurs in the Middle East, Mediterranean region, and Asia and often occurs in the third decade of life [6]. Although the etiology is uncertain, it is said that the interaction of genetic background with infections agents may occur which lead to altered immune dysregulation with the altered microbiota of the gut and can be identified in patients with Behçet's disease [7].

## 2. Case Presentation

**Figure 1:**
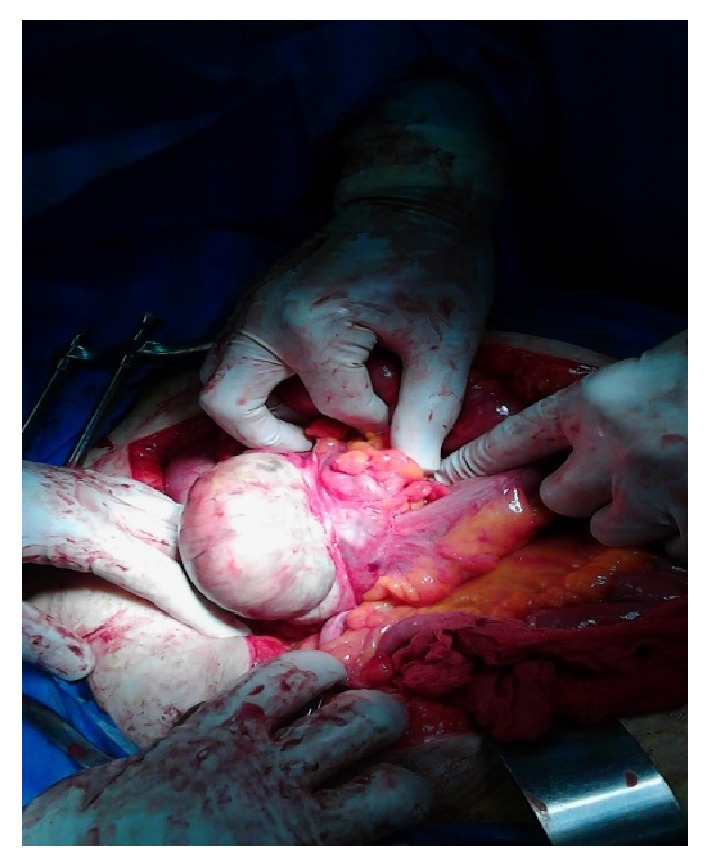
Ischemic field (7 × 5 cm) on the cecal lateral wall.

**Figure 2:**
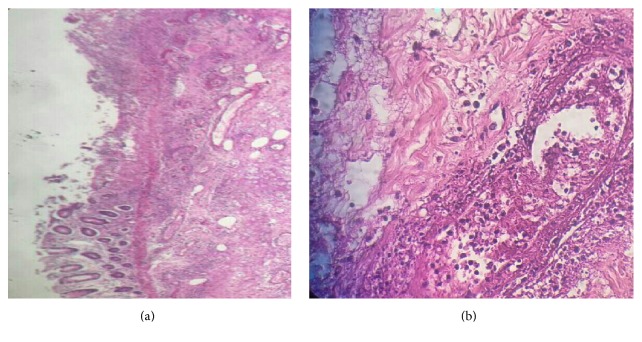
Pathology assessment. (a) Necrotizing inflammation of the mucosa and submucosa. (b) Necrotizing vasculitis.

A 62-year-old female patient was admitted to our clinic with a one-day generalized abdominal pain, slightly more on the right side of the abdomen with dispersion to back accompanied by nausea and vomiting. She complained of not having defecation, gas passing, and appetite. She also complained of chronic constipation, blurred vision, and fever. She had a positive history of type 2 diabetes, hypertension, hyperlipidemia, ischemic heart disease, and cerebrovascular accident and had undergone a coronary artery bypass graft ten years before this admission. Her medications included glibenclamide, losartan, metoral, acarbose, and metformin. At the time of admission, vital signs were as follows: temperature 37.8°C, pulse 130 beats per minute, and blood pressure 125/65 mmHg. On physical examination, she was confused and had acne-like lesions on the face. Patient's abdomen was slightly distended and diffusely tender to palpation, slightly more on the right lower quadrant with rebound but without guarding. Her rectal examination was normal. Laboratory data were as follows: white blood cells (WBC) counts 9700/mm^3^ with 92.3% polymorph nuclear leukocytes and Hb 14.4 g/dL. Biochemistry tests were normal except blood sugar (=514 mg/dL). Electrocardiogram showed normal sinus rhythm with sinus tachycardia. Abdominal sonography revealed dilated bowels with abundant gas. Abdominal CT showed atherosclerotic plaque in the aortoiliac artery and a minor fluid collection in right lower quadrant. With the impression of acute abdomen, the patient underwent midline laparotomy, where the cecum was found to have partial necrosis in the lateral segment with a dimension of 7*∗*5 cm (Figure 1). Regarding this finding, the diagnosis of ischemic colitis was suggested, and a right hemicolectomy and end-to-end ileocolic anastomosis were performed. No polyps, tumor, or diverticular lesion were seen. Microscopic evaluation revealed hemorrhagic infarction with marked neutrophilic necrotizing inflammation involving mucosa, and submucosa. These histopathologic findings confirmed the diagnosis of ischemic colitis. Pathology assessment also reported acute necrotizing inflammation of the submucosa and necrotizing vasculitis of medium and small vessels (Figure 2). Since we could not find any reason for the recent report and regard patient's vision problem and skin lesions, rheumatologic tests were requested which were positive for HLAB5 and HLAB51 suggestive of Behçet's disease. The patient was discharged twelve days after surgery.

## 3. Discussion

Ischemic colitis, especially left colon, is the most common form of gastrointestinal ischemia [2]. Therefore, ischemic colitis involving the right colon is an infrequent occurrence while ischemic colitis involving only the cecum is extremely rare, as shown by Scharff et al. [8].

Isolated cecal necrosis may develop as a result of atherosclerotic or thromboembolic occlusion of the cecal artery. Our patient also had a history of cerebral infarction, strongly suggesting the presence of atherosclerosis. A small artery disease resulting in colonic ischemia is typically seen in a patient with diabetes mellitus or vasculitis (like our patient) or in patients who have previously undergone radiation therapy [9].

Many authors have reported a highly significant association of HLAB5 and Behçet's diseases [10] and the strong association of Behçet's diseases with HLA-B51 in several ethnic groups is well known [11].

Colonic ischemia may result from high-grade obstruction produced by distal neoplasms and sigmoid volvulus [9]. Our patient's microscopic finding showed no tumor or polyp. It has been argued that nonocclusive cecal necrosis develops in relation to open heart surgery, chronic heart disease, and hemodialysis [3, 4].

In cecal necrosis CT scan may reveal thickening of the cecal wall, intramural bleeding, focal or diffused increase in intestinal diameter, mesenteric arterial thrombus, intestinal pneumatosis, portal or mesenteric venous gas, pneumoperitoneum, and intraabdominal free fluid [9] Abdominal spiral CT scan in our patient only reported a little fluid collection in right lower quadrant.

Cecal necrosis often occurs in elderly patients [12]. Only one of 18 patients of Abel et al. [13] was under 50 years old. Guttormson and Bubrick [14] reported an average age of 68 years for cecal necrosis in their study.

Cecal necrosis is presented with right lower quadrant pain, nausea, vomiting, fever, and leukocytosis, imitating acute appendicitis. Although our patient had nausea, vomiting, and right lower quadrant pain, she had a low-grade fever with no leukocytosis.

If the clinical history and evaluation of the patient direct the physician to suspect cecal necrosis, the patient needs to have emergency surgery. Laparoscopy may be performed for diagnosis and treatment, and laparoscopic partial cecal resection may be performed in appropriate cases [15]. But in the recent case, due to respiratory and cardiopulmonary problems, the relative distension of the abdomen, and also intolerance of pneumoperitoneum, laparoscopy was not possible. Mortality related to cecal necrosis is not seen in most studies [3, 5] and is reported in a few studies [16]. Our patient survived after right hemicolectomy.

## 4. Conclusion

Isolated cecal necrosis should be considered in the differential diagnosis in patients with acute right lower quadrant pains, especially in elderly patients with chronic diseases. It is more frequently seen in patients with heart failure and chronic renal failure. Abdominal pain can indicate a disease with vascular involvement like Behçet's disease, especially in the presence of other clinical findings suggestive of the disease, such as blurred vision and skin lesions. So rollout of these diseases seems necessary. Radiological findings might not be specific in isolated cecal necrosis, but cecal wall thickening that is noted in abdominal CT scan is very diagnostic.
